# Supplementary material for: Driving Factors Influencing the Decision to Purchase Plant-Based Beverages: A Sample from Türkiye
Source: Foods. 2024 Jun 4;13(11):1760. doi: 10.3390/foods13111760 (PMC11172123; doi:10.3390/foods13111760)
Supplement: Supplementary file 1 [file foods-13-01760-s001.zip › Supplementary File S1-Questionnaire Used in the Study.pdf]

Supplementary File S1

Questionnaire Used in the Study-English Version

1. Your gender:
   - o Woman
   - o Man
2. Your educational level:
   - o Primary school
   - o Secondary school
   - o High school
   - o Bachelor degree
   - o Master’s and Doctorate
3. Your occupation:
   - o Housewife
   - o Private sector
   - o Civil servant
   - o Student
   - o Retired
   - o Self-employment
   - o Unemployed
4. Your marrital status:
   - o Married
   - o Single
5. Your income:
   - o Below the minimum wage
   - o Above the minimum wage
   - o 11.000-22.000 TRY
   - o 22.000- 33.000 TRY
   - o 33.000 TRY and above
   - o I am a student and get pocket money from my parents
6. What is your year of birth?

   …………………….
7. Your height:

   …………………….
8. Your weight:

……………………

9. Do you have lactose intolerance?
    - Yes
    - No
    - I am not sure
10. Do you suffer from bloating/gas when you drink cow's milk (or sheep's or goat's milk)?
    - Yes
    - No
    - Sometimes
11. Which one describes your dietary pattern?
    - I eat a mixed diet based on animal and plant foods.
    - I am vegetarian
    - I'm vegan
    - I'm a pescatarian
    - Other
12. Have you had any new illness diagnosed by a doctor in the last 12 months?
    - Yes
    - No
13. Please tick any new disease(s) diagnosed by a doctor in the last 12 months.
    - Obesity
    - High Blood Pressure
    - Musculoskeletal diseases
    - Hormonal diseases
    - Cancer
    - Cardiovascular diseases
    - Diabetes
    - Respiratory system diseases
    - Digestive system diseases (liver, gall bladder, stomach, etc.)
    - Vitamin and mineral deficiencies (iron, vitamin B12 deficiency, etc.)
    - Mental problems (depression, overeating, vomiting, night eating, etc.)
    - High blood lipids (LDL cholesterol, total cholesterol, triglycerides, etc.)
    - Other:
14. How many people live at home, including you (excluding animal friends)?

……………………

15. Which of the following describes you?
    - o I live alone
    - o I live with spouse/partner and we have no children
    - o I live with spouse/partner and we have a child
    - o I am not married and live with family members
    - o I live with roommates
    - o I am single and live with my child/children
16. Has anyone in your household had a baby in the last 12 months (It can be your baby or the baby of a family member you live with)
    - o Yes
    - o No
17. Have you changed your city of residence in the last 12 months?
    - o Yes
    - o No
18. Have you had a occupation change in the last 12 months?
    - o Yes
    - o No
19. Which of the following plant-based beverages have you consumed before?
    - o Soy based beverage
    - o Almond based beverage
    - o Walnut based beverage
    - o Rice based beverage
    - o Oat based beverage
    - o Corn based beverage
    - o Coconut based beverage
    - o Hazelnut based beverage
    - o Pistachio based beverage
    - o I haven't consumed any of them
20. What describes your eating habits in the last 12 months?
    - o My eating habits have changed positively
    - o My eating habits have changed negatively
    - o No change in my eating habits
21. Have you personally purchased plant-based beverage in the last month?

- No
- Yes I bought 1 time last month
- Yes I bought 2 times last month
- Yes I bought 3 times last month
- Yes, I regularly buy plant-based milk

Consumers' Plant-Based Beverage Consumption

	Strongly Agree	I agree	I am undecided	I disagree	Strongly Disagree
I exercise regularly.					
Healthy eating is important to me.					
Plant-based milk is healthier than animal milk.					
Plant-based beverages are environmentally friendly.					
If I want to buy plant-based beverage, I can buy it.					
What is important to me is that many people pay attention to a healthy diet.					
Many people who are important to me think that plant-based milk is healthy.					
What matters to me is that many people consider plant-based beverages to be environmentally friendly.					

Importantly for me, many people pay attention to environmentally friendly nutrition.					
I trust the label information of plant-based beverages.					
I trust the label information of organic plant-based beverages.					
It is too expensive for me to buy plant-based beverage.					
When people interfere with natural processes, it often has devastating consequences.					
The environment is heavily exploited by humans.					
Plants and animals have the same right to exist as humans.					
The balance of nature is strong enough to cope with the effects of modern industrial nations (R).					
I would like to find plant-based beverages wherever I shop or eat.					
I would like to see plant-based beverages available online.					
I would have more money to buy more healthy and					

environmentally friendly foods.					
I would like to learn more about the environmental and health aspects of different foods.					
The taste of cow's milk I don't like the taste of cow's milk.					
I like the taste of plant-based beverages.					
Plant-based foods are healthier than animal-based foods.					
Plant-based beverages are healthier than animal-based milks.					
I do not trust producers of plant-based beverage (R).					
I prefer to buy plant-based beverage, even if it is expensive.					
There is not enough variety of plant-based beverage available in stores/markets/markets.					
The taste of a food/drink is more important to me than its impact on the environment (R).					

Consumers' Preferences For Plant-Based Beverages

	Strongly Agree	I agree	I am undecided	I disagree	Strongly Disagree
I usually buy the same brand.					
I buy vegetable-based beverage with no added sugar.					
I buy plant-based beverage with flavors (e.g. strawberry, cocoa).					
I buy plant-based beverage fortified with calcium.					
I buy low-calorie plant-based beverage.					
I buy high-protein plant-based beverage.					
I buy low-fat vegetable-based beverage.					
I buy pasteurized vegetable-based beverage.					
I buy low-priced vegetable-based beverage.					
I buy plant-based beverage that I have tasted before.					
I buy plant-based beverage on promotion					

(such as 2 for 1).					

Factors İnfluencing The Decision To Purchase Plant-Based Beverage

	Strongly Agree	I agree	I am undecided	I disagree	Strongly Disagree
Brand					
Taste					
Freshness					
Nutritional value					
What plant is it made from?					
Price					
Health benefits					
Whether there was a promotion or not					

22. How likely are you to buy plant-based milk next month?

- o None
- o It's unlikely
- o Maybe I'll take it.
- o I'll probably get it
- o I will definitely buy it
