# Supplementary material for: Driving Factors Influencing the Decision to Purchase Plant-Based Beverages: A Sample from Türkiye
Source: Foods. 2024 Jun 4;13(11):1760. doi: 10.3390/foods13111760 (PMC11172123; doi:10.3390/foods13111760)
Supplement: Supplementary file 1 [file foods-13-01760-s001.zip › Supplementary Tables S2 and S3.pdf]

Supplementary Table S1. Discriminant Validity of Study Variables

	CR	AVE	MSV	ASV	HC	KN	SN	PP	PI	ABB	ATT	TR	EP	AW	AVA
**HC**	0.931	0.730	0.004	0.002	**0.990**										
**KN**	0.872	0.696	0.672	0.364	0.027	**0.853**									
**SN**	0.903	0.757	0.623	0.342	0.008	0.820	**0.789**								
**PP**	0.880	0.715	0.325	0.195	-0.070	0.592	0.571	**0.419**							
**PI**	0.938	0.793	0.539	0.340	0.011	0.820	0.789	0.570	**0.791**						
**ABB**	0.949	0.862	0.555	0.072	-0.028	0.042	0.037	0.039	-0.003	**0.799**					
**ATT**	0.931	0.821	0.067	0.066	0.036	-0.014	-0.018	-0.007	-0.056	0.745	**0.702**				
**TR**	0.915	0.636	0.429	0.283	0.065	0.742	0.713	0.511	0.719	-0.071	-0.111	**0.662**			
**EP**	0.904	0.761	0.255	0.174	-0.060	0.520	0.500	0.370	0.485	0.315	0.258	0.411	**0.427**		
**AW**	0.887	0.669	0.460	0.313	-0.003	0.768	0.739	0.537	0.734	0.133	0.075	0.655	0.505	**0.704**	
**AVA**	0.912	0.775	0.436	0.292	0.022	0.731	0.702	0.509	0.694	0.197	0.139	0.615	0.504	0.678	**0.660**

HC: Health Consciousness; KN: Knowledge; SN: Subjective Norm; PP: Perceived Price; PI: Purchase Intention; ABB: Actual Buying Behaviour; ATT: Attitude; TR: Trust; EP: Environmental Protection; AW: Animal Welfare; AVA: Availability; CR: Composite Reliability, AVE: Average Variance Extracted; MSV: Maximum Shared Variance; ASV: Average Shared Variance

Supplementary Table S2. Correlation coefficients between study variables

		**HC**	**KN**	**SN**	**PP**	**TR**	**EP**	**AW**	**AVA**	**ATT**	**PI**
**KN**	**s**	0.020									
	**p**	0.550									
**SN**	**s**	0.001	**0.782**								
	**p**	0.987	**<0.001*** **								
**PP**	**s**	-0.018	**0.514**	**0.413**							
	**p**	0.586	**<0.001*** **	**<0.001*** **							
**TR**	**s**	0.051	**0.624**	**0.628**	**0.399**						
	**p**	0.117	**<0.001*** **	**<0.001*** **	**<0.001*** **						
**EP**	**s**	-0.005	**0.467**	**0.474**	**0.244**	**0.260**					
	**p**	0.874	**<0.001*** **	**<0.001*** **	**<0.001*** **	**<0.001*** **					
**AW**	**s**	0.001	**0.703**	**0.743**	**0.349**	**0.516**	**0.547**				
	**p**	0.966	**<0.001*** **	**<0.001*** **	**<0.001*** **	**<0.001*** **	**<0.001*** **				
**AVA**	**s**	0.014	**0.644**	**0.634**	**0.382**	**0.536**	**0.451**	**0.607**			
	**p**	0.660	**<0.001*** **	**<0.001*** **	**<0.001*** **	**<0.001*** **	**<0.001*** **	**<0.001*** **			
**ATT**	**s**	0.009	0.033	-0.004	0.012	-0.027	**0.086**	**0.067**	0.055		
	**p**	0.787	0.313	0.899	0.712	0.414	**0.009** **	**0.042***	0.091		
**PI**	**s**	0.021	**0.794**	**0.667**	**0.607**	**0.681**	**0.404**	**0.570**	**0.599**	0.014	
	**p**	0.528	**<0.001*** **	**<0.001*** **	**<0.001*** **	**<0.001*** **	**<0.001*** **	**<0.001*** **	**<0.001*** **	0.672	
**ABB**	**s**	-0.026	0.034	0.042	0.056	-0.036	**0.266**	**0.110**	**0.236**	**0.537**	0.032
	**p**	0.423	0.296	0.196	0.085	0.277	**<0.001*** **	**0.001** **	**<0.001*** **	**<0.001*** **	0.325

*HC: Health Consciousness; KN: Knowledge; SN: Subjective Norm; PP: Perceived Price; PI: Purchase Intention; ABB: Actual Buying Behaviour; ATT: Attitude; TR: Trust; EP: Environmental Protection; AW: Animal Welfare; AVA: Availability; s: Spearman Correlation Coefficient; *p<0,05; **p<0,01; ***p<0,00*
